# Influence of Social and Demographic Factors on Retinoblastoma Outcomes in the United States: A Systematic Review

**DOI:** 10.1002/cesm.70081

**Published:** 2026-05-16

**Authors:** Vijay Joshi, Daniel Shaughnessy, Natalia Dellavalle, Louis Leslie, Michael Edwards, Sandra Luna‐Fineman, Timothy Waxweiler, Tianjing Li, Riaz Qureshi

**Affiliations:** ^1^ Anschutz Medical Campus University of Colorado Aurora Colorado USA

**Keywords:** cancer, ocular neoplasms, retinoblastoma, social determinants of health

## Abstract

**Introduction:**

Social determinants of health (SDOH), which include economic stability, education access and quality, community and social context, healthcare access and quality, and neighborhood and built environment, are known to be related to variation in health outcomes across a variety of health conditions. Ocular neoplasms are among the health conditions that have been shown to be affected by SDOH, and retinoblastoma is the most prevalent form in children. Our objective was to systematically review documented associations between SDOH and retinoblastoma in the US.

**Methods:**

We followed a pre‐established protocol. We searched Medline, Embase, and Web of Science from 2000 to November 2023 using a mix of keywords and controlled vocabulary. We included primary studies of any design that evaluated one or more relationships between SDOH and retinoblastoma outcomes, such as survival, mortality, disease staging, types of care, and incidence. We extracted data on study design, population characteristics, SDOH domains and indicators, and estimates of associations with retinoblastoma outcomes. We assessed risk of bias using a modified Newcastle‐Ottawa Scale and performed narrative syntheses.

**Results:**

We included 26 studies that had reported a total of 552 associations. Social and community context, notably race and ethnicity, was the most commonly examined domain, suggesting evidence of worse survival outcomes for Black and Hispanic patients. The most clinically and policy‐relevant patterns were concentrated in social and community context, economic stability, and healthcare access and quality SDOH domains, where measures such as race and ethnicity, poverty, unemployment, and insurance status were repeatedly associated with stage at diagnosis, enucleation, and survival.

**Conclusion:**

SDOH were frequently associated with retinoblastoma diagnosis, treatment, and survival in US studies. The most clinically relevant findings relate to insurance‐related access barriers, socioeconomic disadvantage, and social and community factors, while environmental and occupational exposures were more informative for disease cause and prevention. Our findings underscore the need for more consistent SDOH measurement and adjustment methods to improve study comparability and to inform targeted interventions to address retinoblastoma health outcomes.

## Summary

- Retinoblastoma is a rare eye cancer that mainly affects young children. In the United States, most children survive this disease. However, outcomes are not the same for everyone.
- In this study, we reviewed 26 research papers to understand how social and economic factors, such as income, education, race, insurance, and where a child lives, affect diagnosis and treatment. We identified over 500 links between these factors and important outcomes, including how early the cancer was detected, whether the eye could be saved, and survival.
- Overall, children from lower‐income families, rural areas, and racial and ethnic minority groups tended to have worse outcomes. We also found that environmental exposures, such as air pollution and pesticides, may increase the risk of developing retinoblastoma.
- These findings show that social and economic conditions can strongly influence health, even for a cancer with high survival rates. Improving access to care and providing more support for underserved communities could help reduce these differences.

## 1 Introduction

Investigating the influence of social determinants on different health states is critical to addressing upstream causes of health outcomes in populations as well as providing optimal care for individual patients [1, 2]. The United States (US) Department of Health and Human Services defines social determinants of health (SDOH) as “the conditions in the environments in which people live, learn, work, play, worship, and age.” [3] In alignment with Healthy People 2030, SDOH encompass five key domains: economic stability, education access and quality, healthcare access and quality, neighborhood and built environment, social and community context [1].

Retinoblastoma, the most common childhood ocular cancer, represents 3% of all childhood cancers [4, 5]. Retinoblastoma is broadly categorized as unilateral, most often due to somatic mutations, or bilateral, most often due to germline mutations [6]. Due to these different etiologies, unilateral retinoblastoma is often regarded as nonheritable and bilateral retinoblastoma as heritable retinoblastoma. Modern treatments typically follow a “save the life, globe, sight” paradigm, which aims to balance survival versus unnecessary enucleations to favor globe‐salvaging techniques [7]. This paradigm has resulted in 99% survival to adulthood of children in developed countries with retinoblastoma [8]. Our objective was to assess how SDOH affect the development, detection, treatment, and prognosis of retinoblastoma in the US through a systematic review. Because the US evidence base is scattered across registry analyses, treatment studies, and causal studies of environmental and occupational exposures, a systematic synthesis is needed to clarify which SDOH are most consistently associated with retinoblastoma outcomes and which may be most actionable for clinical and policy intervention in the US.

## 2 Methods

We performed this review within a suite of systematic reviews undertaken by Cochrane Eyes and Vision US Project (CEV@US) to examine various aspects of eye health and their relation to the SDOH [9]. In this review, we focused on retinoblastoma and followed a protocol published on Open Science Framework [10]. None of the authors of this review were authors of any of the included primary studies. Appendix A presents a detailed description of methods, summarized below.

We identified eligible studies from a master database developed and maintained by CEV@US on social determinants of ocular health in the US. The master database was constructed by searching MEDLINE, Embase, and Web of Science Core Collection from year 2000 to present. We screened full texts of the records tagged for ocular neoplasms for relevance to retinoblastoma. We also searched the reference list of included studies. We included primary studies that examined the relationship between SDOH–defined by the Healthy People 2030 framework [1] and retinoblastoma. We did not restrict studies based on their design.

We extracted characteristics of the study, population, exposures, outcomes, and estimates of the associations using Systematic Review Data Repository (SRDR + ) and Qualtrics [11, 12]. We assessed risk of bias using a modified Newcastle‐Ottawa Scale. For both data extraction and risk of bias we applied a single assessor plus verification approach, with discrepancies resolved through discussion or adjudication with a third reviewer.

Due to the heterogeneity of the studies and analyses, including the definitions of exposures, outcomes, and measures of association, we were unable to conduct any meta‐analyses. Anticipating this limitation from prior experience with SDOH reviews, we present a narrative synthesis of the associations organized by SDOH domain. We created data displays to portray the breadth and types of associations.

We further classified association estimates according to their directionality and statistical significance [13]. Association directionality was classified as follows: favorable if a “worse” exposure, compared with “better” exposure, was associated with improved outcomes (e.g., reduced incidence); unfavorable if a “worse” exposure was linked to worse outcomes (e.g., increased mortality); and null if no clear relationship was observed. For example, if a study found that lower income levels were associated with increased mortality when compared with higher income levels, we classified this as unfavorable. In some cases, the directionality of the exposure‐outcome relationship could not be determined, such as when there was no clearly defined reference group, and these associations were labeled “NA”. Given the large number of associations across studies, we could not describe all estimates in text when synthesizing the evidence. Instead, we highlighted those of substantial effect size and focused on summarizing trends and findings against expected trends, regardless of statistical significance.

## 3 Results

**Figure 1:**
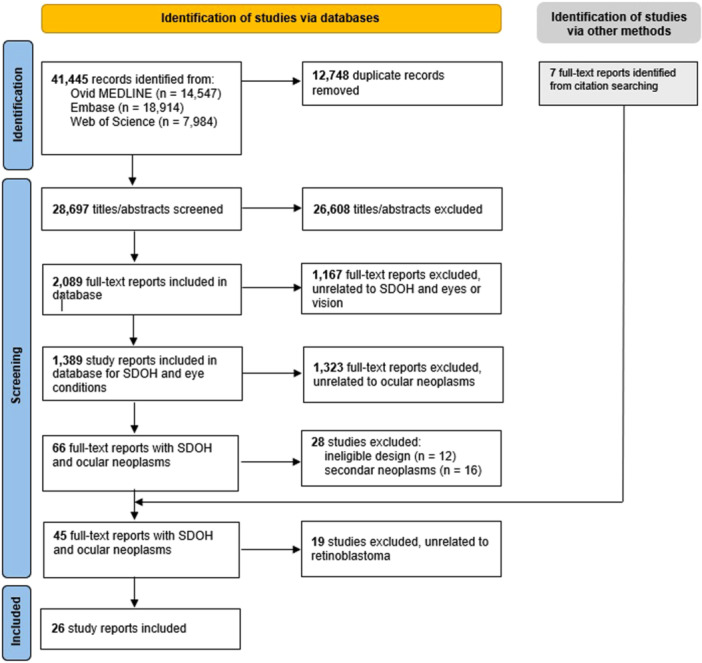
PRISMA flowchart for systematic review.

As of November 30, 2023 the master database contained 1,389 reports related to SDOH and eye health, 66 of which were categorized under ocular neoplasms and were further assessed for this review. We included 38 records from the database and identified 7 additional reports through searching the reference list of included studies. Out of the 45 eligible studies related to ocular neoplasms, 26 described associations with retinoblastoma, which are included in this review (Figure 1). The remaining 19 (plus two from this review that cover multiple cancers) will be included in a separate review on the associations between SDOH measures and non‐retinoblastoma ocular cancer [14].

### 3.1 Characteristics of Included Studies

A total of twelve case‐control studies and 14 cohort studies published after the year 2000 were included; data for the studies were collected from 1969 to 2021 (Table 1). Most studies were based on secondary data analyses of previously established cancer registries and databases, including the SEER (Surveillance, Epidemiology, and End Results Program) (*n* = 9), Children's Oncology Group (*n* = 4), and the California Cancer Registry (*n* = 4). There are two studies each from the Texas Cancer Registry and a multi‐center North American study. Also included is one study each from the National Cancer Database, Alaska Native Tumor Registry, International Agency for Research on Cancer registries, the NIH‐AARP Diet and Health Study cohort, St. Jude Children's Research Hospital, and St. Louis Children's Hospital. One study compared data from St Jude Children's Research Hospital to data from SEER, so it is represented twice in the preceding summary.

**Table 1:** Summary of selected characteristics of included studies.

Study	Study Years	Study Design	Study Population (Representativeness)	Sample Size	Participants' Characteristics	SDOH Domain Studied (Role)	Ocular Neoplasm Measure (Role)	Study Findings	Funding Source
Abdolahi 2013	1998–2006	Case‐control study	Patients diagnosed as having sporadic bilateral retinoblastoma from January 1998 to May 2006 and treated at one of the nine participating institutions in the USA and Canada (national)	198 cases; 245 controls	Average father's age at conception (SD): 32.7 (6.2) Female children: 49% White: 52% Hispanic: 10%	Economic stability (exposure)	Retinoblastoma ‐ incidence (outcome)	This study suggested a potential role of welding metals and pesticide exposure to parents resulting in retinoblastoma, but their results did not meet statistical significance.	US National Institutes of Health
Alfaar 2022	1973–2017	Cohort study	Individuals diagnosed as having uveal melanoma between 1973 and 2017 and identified in the SEER 18 database (national)	10,557 cases	Age group 0–39: 8% 40–59: 35% 60–80+: 57% Female: 48% White: 98% Hispanic: NR	Healthcare access and quality; Social and community context (exposure)	Uveal melanoma ‐ survival (outcome)	The study found better 10‐year survival in uveal melanoma patients who maintained their marriages compared to unmarried, divorced, and widowed patients.	None
Bunin 2011	2002–2007	Case‐control study	Children diagnosed as having sporadic bilateral retinoblastoma from January 1998 to May 2006 at nine institutions in the US and Canada (national)	199 cases; 247 controls	Father's age at child's birth < 25–29: 35% 30–39: 55% 40+: 10% White fathers: 74% Hispanic fathers: 10%	Education access and quality; Economic stability; Social and community context (exposure)	Retinoblastoma ‐ incidence (outcome)	The study found that neither being a race other than non‐Hispanic white nor having a college degree were associated with an increased or decreased odds of developing childhood retinoblastoma when adjusting for birth year and father's education level.	US National Institutes of Health
Carozza 2009	1990–1998	Case‐control study	Texas children born between 1975 and 1998 and less than age 15 years at cancer diagnosis identified from the Texas Cancer Registry (state)	109 cases; 1,802 controls	Average age (SD): NR (NR) Female: 43% White: 46% Hispanic: 39%	Neighborhood and built environment (exposure)	Retinoblastoma ‐ incidence (outcome)	The study found no association between parental agriculture‐related exposures and the odds of childhood retinoblastoma.	US National Institutes of Health and National Cancer Institute
Cheung 2013	1973–2009	Cohort study	Patients diagnosed with retinoblastoma between 1972 and 2009 identified in the SEER database (national)	1,456 cases	Patient age < 20: 99% ≥ 20: 1% Female: 48% White: 73% Hispanic: NR	Economic stability; Education access and quality; Healthcare access and quality; Social and community context (exposure)	Retinoblastoma ‐ specific mortality (outcome)	The study found mixed results with no association between income and retinoblastoma‐specific mortality, but increased hazard of overall mortality depending on rurality and race/ethnicity.	NR
Choudhry 2023	2006–2017	Cohort study	Patient diagnosed as having primary uveal melanoma, conjunctival melanoma, or retinoblastoma between January, 2006 and December, 2017, treated with curative intent, and identified in the National Cancer Database (national)	4,800 cases	Median age at diagnosis: 64 Female: 47% White: 92% Hispanic: NR	Social and community context, Healthcare access and quality, Economic stability (exposure)	Uveal melanoma, conjunctival melanoma, retinoblastoma ‐ advanced clinical tumor presentation (outcome) and 5‐year survival (outcome)	The study did a combined analysis but found having insurance and lower income were both significantly associated with worse ocular cancer staging on presentation.	Research to Prevent Blindness Inc. and the Wilmer Eye Institute
Emmanuel 2012	1995–2006	Cohort study	Patients diagnosed as having ocular cancer from 1995 to 2006 from six US states and two metropolitan areas (national)	178 cases	Age ≤ 60: 36% Age > 60: 64% Female: 40% White: 91% Hispanic: 2%	Social and community context (exposure)	All eye neoplasms ‐ incidence (outcome)	The study found no differences between incidence rates of eye cancers among different races.	Intramural Research Program of the National Institutes of Health and the National Cancer Institute
Fei‐Zhang 2023	1975–2017	Cohort study	Patients greater than 18 years old diagnosed as having retinoblastoma between 1975 and 2017 and identified in the SEER database (national).	1,975 cases	Age 0–9: 44% Age 10–19: 56% Female: 50% White: 61% Hispanic: 20%	Economic stability; Education access and quality; Healthcare access and quality; Neighborhood and built environment; Social and community context (exposure)	Retinoblastoma ‐ advanced staging on preliminary presentation (outcome) and primary surgery receipt (outcome)	This study found greater vulnerability in multiple composite social vulnerability indexes was associated with more advanced disease at diagnosis.	NR
Friedrich 2017	2000–2010	Case‐control study	Children and adolescents (age 0–19 years) diagnosed with extracranial embryonal tumors between 2000 and 2010 and identified in the SEER database (national)	824 cases	Average age (SD): NR (NR) Female: NR White: 53% Hispanic: 28%	Social and community context (exposure)	Retinoblastoma ‐ incidence (outcome)	This study found that from 2000 to 2010, Hispanics had a higher rate of developing retinoblastoma compared to whites for both overall incidence and specifically bilateral disease.	Hyundai Hope on Wheels Scholar Grant
Ghosh 2013	1988–2008	Case‐control study	All cancer cases among all children aged 0–5 years who were diagnosed with cancer from 1988 to 2008 and listed in the California Cancer Registry (state)	4,015 cases; 80,658 controls	Mother's age at childbirth < 20: 26% 20–34: 75% ≥ 35: 14% Female children: 49% Mother's race/ethnicity ‐ White: 26% Mother's race/ethnicity ‐ Hispanic: 56%	Neighborhood and built environment (exposure)	Retinoblastoma ‐ incidence (outcome)	This study found that maternal exposure to nitric oxide (a chemical commonly attributed to pollution and traffic exhaust) during the third trimester increased the odds of childhood bilateral retinoblastoma.	Environmental Health Sciences and the National Cancer Institute at the National Institutes of Health
Green 2016	2005–2010	Cohort study	Children with unilateral retinoblastoma who had undergone unilateral enucleation for advanced intraocular disease from 2005 to 2010 at a Children's Oncology Group institution (national)	203 cases	Average age (SD): NR (NR) Female: NR White: 60% Hispanic: 32%	Economic stability; Healthcare access and quality; Social and community context, Education access and quality (exposure)	Retinoblastoma ‐ high risk histopathologic features (outcome)	This study found mixed evidence of an effect of income on high‐risk pathologic features of retinoblastoma but a significant association with race/ethnicity.	None
Guo 2023	2004–2015	Cohort study	Patients less than 19 years diagnosed with retinoblastoma from 2004 to 2015 identified in the SEER database (national)	923 cases	Age 0: 43% Age 1–4: 53% Age 5–19: 4% Female: 51% White: 75% Hispanic: NR	Economic stability; Healthcare access and quality (exposure)	Retinoblastoma‐ overall survival (outcome)	This study found no statistically significant differences in overall survival of retinoblastoma between patients of middle vs high income nor patients who lived rural vs urban settings.	Henan Natural Science Foundation of China
Heck 2015	1990–2007	Case‐control study	Children younger than age 6 diagnosed as having retinoblastoma and living within 5 miles of an air pollution monitor in California between 1990 and 2007 and identified in the California Cancer Registry records (state)	30,704	Father' age < 29: 50% 30–34: 25% 35+: 25% Mother's race/ethnicity White: 25% Hispanic: 53% Female children: 49%	Neighborhood and built environment (exposure)	Retinoblastoma ‐ incidence before age 6 (outcome)	This study found associations between maternal exposure to multiple types of chemicals during pregnancy and the likelihood of offspring developing retinoblastoma in the first year of life and being diagnosed before the age of 6.	US National Institutes of Health
Heck 2016	2007–2013	Case‐control study	Children younger than age 6 diagnosed as having cancer between January 2007–September 2013 and identified in the California Cancer Registry records (state)	125 cases	Average age (SD): NR (NR) Female: NR White: NR Hispanic: NR	Neighborhood and built environment (exposure)	Retinoblastoma ‐ incidence before age 6 (outcome)	This study found a positive association between the odds of bilateral childhood retinoblastoma and parental exposure to butadiene, chromium, nickel, and maternal smoking during pregnancy.	US National Institutes of Health, University of California Tobacco‐Related Disease Research Program, Cancer Prevention Institute of California, University of Southern California, Public Health Institute, and the California Department of Public Health
Holmes 2023	2000–2017	Cohort study	Children (< 19 years) diagnosed as having retinoblastoma between 2000 and 2017 and identified in the SEER database (national)	439 cases	Age < 1: 28% Age 1–4: 67% Age 5–9: 4% Age 10–14: 1% Female: 43% White: 78% Hispanic: NR	Social and community context; Economic stability (exposure)	Retinoblastoma ‐ survival (outcome) and mortality (outcome)	This study found that there was a sex differential in survival with excess risk of dying identified among males relative to females, which may be explained in part by male X‐linkage.	None
Krishna 2009	1993–1997	Cohort study	Patients from San Francisco, Los Angeles, and New Mexico diagnosed as having retinoblastoma from 1993 to 1997 and identified in the International Agency for Research on Cancer database (national)	1,366 cases	Average age (SD): NR (NR) Female: NR White: NR Hispanic: NR	Social and community context (exposure)	Retinoblastoma ‐ incidence (outcome)	This study did not find differences in the rate ratios between white and Hispanic populations in the US when using pooled data from a Los Angeles, San Francisco, and New Mexico.	The EyeCare Foundation
Lanier 2003	1969–1996	Cohort study	Alaska Natives (less than age 20) diagnosed with cancer from 1969 to 1996 and identified in the Alaska Native Tumor Registry (state)	131 cases	Average age (SD): NR (NR) Female: 46% White: 99% Hispanic: NR	Social and community context (exposure)	Retinoblastoma ‐ incidence (outcome)	This study found that Alaska Native and New Mexican American‐Indian children are statistically more likely to develop retinoblastoma compared to US whites.	The National Cancer Institute
Omidakhsh 2017	2006–2011	Case‐control study	Children diagnosed with sporadic retinoblastoma between July 1st, 2006 and June 30th, 2011 who were diagnosed and/or treated at a Children's Oncology Group Institution or at the Wills Eye Institute (national)	155 cases; 148 controls	Mother's age, ≤ 29: 46% 30–34: 36% 35+: 18% Father's age, ≤ 29: 32% 30–34: 35% 35+: 33% Female children: 51% Mother's race, White: 74% Father's race, White: 70% Mother's ethnicity, Hispanic: 14% Father's ethnicity, Hispanic: 16%	Neighborhood and built environment (exposure)	Retinoblastoma ‐ incidence (outcome)	This study found that parental pesticide use and having professional lawn or landscape services were both statistically associated with increased odds of unilateral disease incidence.	The National Institute of Health, National Cancer Institute, and the National Institute of Environmental Health Sciences
Omidakhsh 2018	2006–2011	Case‐control study	Children diagnosed with sporadic retinoblastoma between June 1st, 2006 and June 30th, 2012 who were diagnosed and/or treated at a Children's Oncology Group Institution or at the Wills Eye Institute (national)	282 cases; 155 controls	Father's age, < 25: 9% 25–39: 80% 40+: 11% Father's race, White: 65% Father's ethnicity, Hispanic: 18%	Economic stability (exposure)	Retinoblastoma ‐ incidence (outcome)	This study found that paternal characteristics such as occupational exposures 10 years prior conception and being older than 30 years old were associated with increased incidence of bilateral retinoblastoma.	The National Institute of Health, National Cancer Institute, National Institute of Environmental Health Sciences, ARRA supplement, Southern California NIOSH Education and Research Center, Centers for Disease Control, and the Jonsson Cancer Center Foundation
Pui 2012	1992–2007	Cohort study	Children diagnosed with multiple types of cancer at St Jude Children's Research Hospital in Memphis, TN between 1962 and 1992 (national)	483 cases	Average age (SD): NR (NR) Female: NR White: 75% Hispanic: NE	Social and community context (exposure)	Retinoblastoma ‐ survival (outcome)	This study found worse 5‐year survival for Black patients with retinoblastoma compared to white patients.	The US National Cancer Institute, American Lebanese and Syrian Associated Charities, and Cheng Cheng and Sigma Tau Pharmaceuticals
Rajeshuni 2019	2000–2014	Cohort study	Retinoblastoma patients aged 18 and under diagnosed between 2000 and 2014 and identified in the SEER 18 database (national)	959 cases	Age at diagnosis < 1: 41% 1–2: 45% > 2: 14% Female: 47% White: 75% Hispanic: 36%	Social and community context (exposure)	Retinoblastoma ‐ enucleation (outcome)	This study found greater odds of enucleation therapy for retinoblastoma in minority populations compared to white and non‐Hispanic populations as well as in the lowest SES tertile compared to the highest SES tertile.	None
Reynolds 2021	1995–2018	Cohort study	All children whose retinoblastoma was diagnosed at St. Louis Children's Hospital from 1995 to 2018, who were followed since diagnosis, and were 18 years of age and younger at the time of chart review (NR) (institutional)	73 cases	Median age at diagnosis: 10 months Female: NR White: NR Hispanic: NR	Education access and quality (Outcome)	Retinoblastoma diagnosis (exposure)	This study found that children who received chemotherapy treatment for retinoblastoma were at greater odds of self‐reporting school difficulty and needing an individualized education plan compared to those who did not receive chemotherapy.	Research to Prevent Blindness Inc. and the National Center For Advancing Translational Sciences of the National Institutes of Health
Thompson 2008	1990–2002	Case‐control study	All Texas birth records from January 1, 1990 to December 31, 2002 were followed for cancer incidence as reported to the Texas Cancer Registry as of January 1, 2003 (state)	179 cases; controls NR	Average age (SD): NR (NR) Female: NR White: NR Hispanic: NR	Neighborhood and built environment (exposure)	Retinoblastoma ‐ incidence (outcome)	This study looked at various farming chemicals and hazardous air pollutant exposures in Texas and found no difference in the odds of developing retinoblastoma between cases and controls.	National Institutes of Health and the National Cancer Institute
Thompson 2022	1998–2011	Case‐control study	Retinoblastoma cases aged 5 and younger diagnosed from 1988 to 2013 identified in the California Cancer Registry (state)	335 cases; 123,166 controls	Maternal age ≤ 19: 10% 20–34: 74% ≥ 35: 16% Female children: 49% Maternal race/ethnicity White: 29% Hispanic: 51%	Neighborhood and built environment (exposure)	Retinoblastoma ‐ incidence (outcome)	This study found that exposures to various pesticides during pregnancy were associated with higher odds of childhood unilateral retinoblastoma.	US National Institutes of Health and the Alex's Lemonade Stand Pediatric Oncology Student Training Program
Truong 2015	2000–2010	Cohort study	Children aged 0 to 9 years who were diagnosed as having retinoblastoma from January 1, 2000, through December 31, 2010, were selected from the SEER database (national)	830 cases	Age at diagnosis < 1: 40% 1–4: 56% 5–9: 4% Female: 42% White: 73% Hispanic: 33%	Economic stability; Education access and quality; Social and community context (exposure)	Retinoblastoma enucleation (outcome), 5 year relative survival (outcome), risk of advanced disease at diagnosis (outcome)	This study found associations between educational attainment and level of crowding with enucleation, as well as race/ethnicity with enucleation, extraocular retinoblastoma, and survival at 5 years.	NR
Wong 2014	1975–2009	Cohort study	Children 5 and younger diagnosed as having retinoblastoma and identified in the SEER 18 database (national)	721 cases	Age at diagnosis < 1: 43% 1–4: 57% Female: 45% White: 41% Hispanic: 31%	Social and community context (exposure)	Retinoblastoma ‐ incidence (outcome)	This study found a higher incidence rate of bilateral retinoblastoma for White Hispanic males compared to White non‐Hispanics and White Hispanic males compared to White Hispanic females.	Intramural Research Program of the National Institutes of Health and the National Cancer Institute

Abbreviations: NR, not reported; inc, incorporated; SD, standard deviation; SEER, Surveillance, Epidemiology, and End Results; US, United States

Across the 26 included studies, a wide variety of exposures representing all five SDOH domains were investigated, with social and community context being the most frequently examined, primarily through measures of race and ethnicity. Other commonly assessed exposures included economic stability factors (e.g., income and occupational exposures), neighborhood and built environment conditions (e.g., exposure to environmental toxins such as pesticides and tobacco), healthcare access indicators (e.g., insurance type and distance to care), and educational attainment levels. Outcomes primarily focused on survival and mortality, disease staging at diagnosis, treatment type such as enucleation, and the incidence of retinoblastoma. A small number of studies also evaluated how retinoblastoma treatments impacted educational outcomes, highlighting the bidirectional relationship between health and social determinants.

### 3.2 Risk of Bias of Included Studies

**Figure 2:**
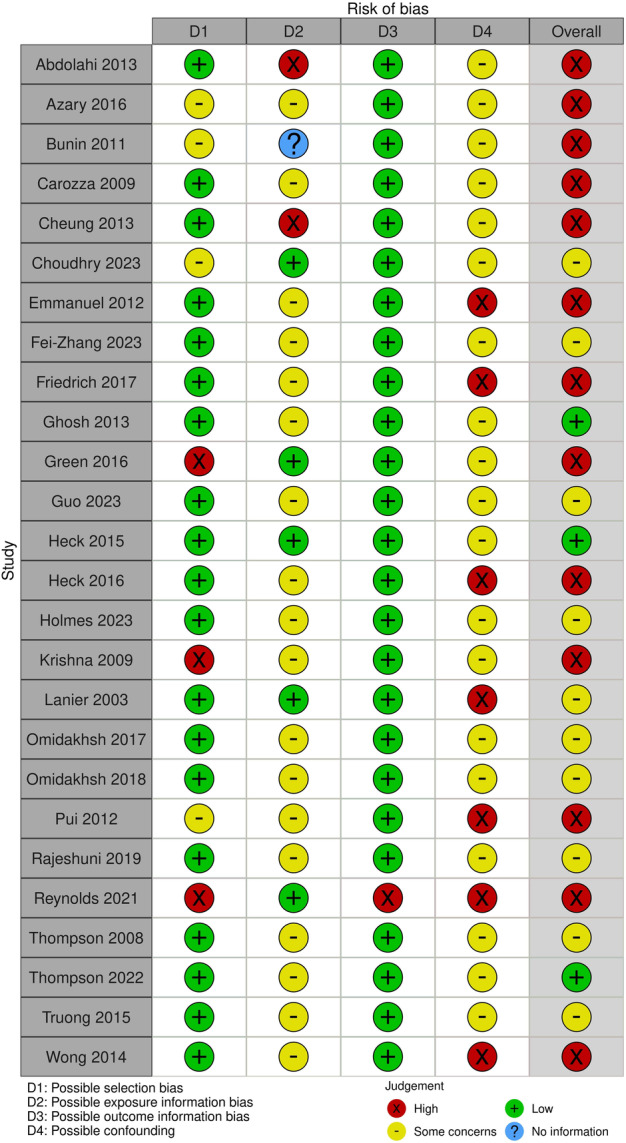
Risk of bias stoplight plot.

Of the 26 included studies, we judged 13 (50%) to have a high risk of bias [15, 16, 17, 18, 19, 20, 21, 22, 23, 24, 25, 26, 27], 10 (38.5%) to have a moderate risk of bias [28, 29, 30, 31, 32, 33, 34, 35, 36, 37], and 3 (11.5%) to have a low risk of bias [37, 39, 40] (Table S1). Figure 2 displays the summary of risk of bias assessments for included studies. Overall, the primary areas of bias concern were participant selection, exposure assessment, and possible confounding of results.

### 3.3 Associations

Across the 26 studies, we found 552 associations between SDOH and a retinoblastoma outcome. Table 2 provides a summary of the overall pattern by showing the number of unfavorable, favorable, null and not applicable associations within each SDOH domain.

**Table 2:** Directionality of associations by SDOH domain.

SDOH domain	Total associations	Directionality of associations (*n* = 552)			
		Unfavorable	Favorable	Null	Not applicable
Economic stability	63	39	19	1	4
Education access and quality	16	13	0	0	3
Healthcare access and quality	9	4	3	2	0
Neighborhood and built environment	292	177	102	13	0
Social and community context	132	82	23	3	24
Multiple domains	27	21	6	0	0
SDOH as the outcome: Education access and quality	13	2	10	1	0

**Figure 3:**
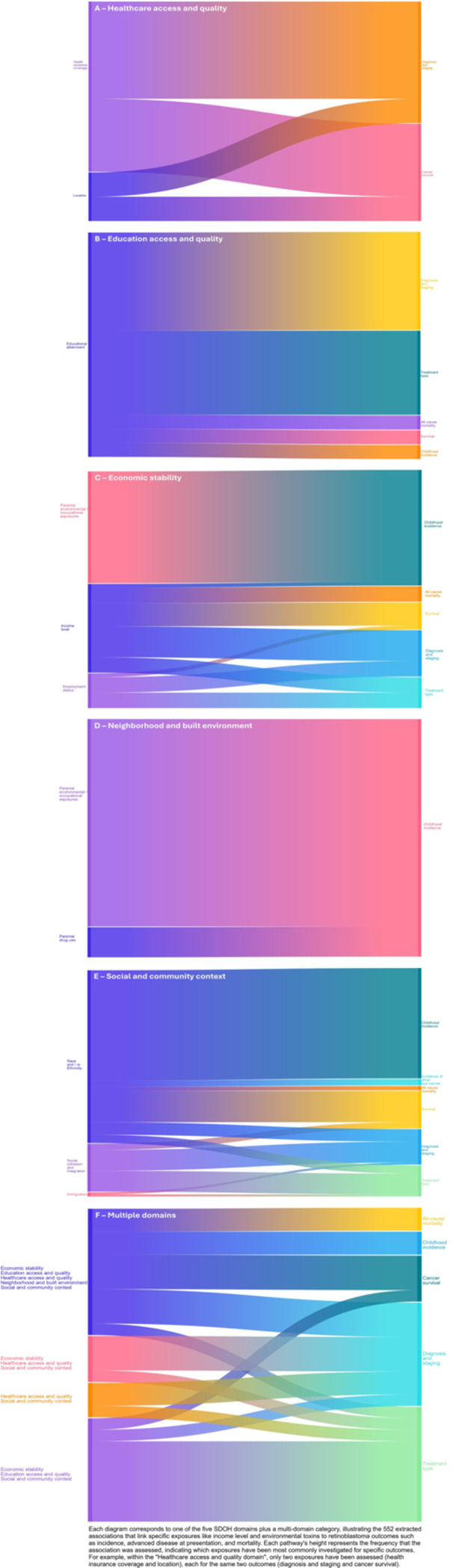
(A–F) Associations between social determinant domains and retinoblastoma outcomes.

Figure 3 serves as a visual overview of how exposures within each domain mapped to specific outcomes. Most domains were assessed through only 1‐3 categories of exposures. These exposures were usually mapped to multiple outcomes. For example, educational attainment was assessed for all five outcome categories of interest (diagnosis and staging, type of treatment received, mortality, survival, and childhood incidence). Some exposures, however, were only assessed for one outcome type; for example, parental environmental and occupational exposures were examined primarily in relation to incidence, which is important for causation and prevention but less directly informative for clinical intervention.

We present narrative summaries of the evidence according to the social determinant domain. Table 2 presents all associations, estimates, domains, and directions of effect for included studies and allows filtering by outcome for further exploration.

### 3.4 Healthcare Access and Quality

Five studies examined nine associations within the domain of healthcare access and quality, covering geographic location and insurance type. Four of the nine associations (44.4%) were categorized as ‘unfavorable’ (associated with worse retinoblastoma outcomes), 3 (33.3%) were ‘favorable’ (related to improved outcomes), and 2 (22.2%) were ‘null’ (no clear relationship). Guo 2023 (moderate risk of bias) found that rural or rural‐urban persons may have longer survival with respective adjusted hazard ratios of 3.43 (0.98, 12.01) and 1.94 (0.54, 7.05) compared with urban residents [30]. Similar relationships were also found between rural versus urban or metropolitan residences in Cheung 2013 (high risk of bias) and Holmes 2023 (moderate risk of bias), although only Cheung 2013 found statistical evidence of an association (*β* = 0.93; *p* = 0.02) [19, 31]. Thompson 2008 (moderate risk of bias) also studied urbanicity by estimating standardized morbidity ratios with observed‐over‐expected incident events, finding evidence suggesting greater incidence in metropolitan than urban locations. However, they did not make direct comparisons between the two [36]. Green 2016 (high risk of bias) studied insurance type and found that individuals with non‐private insurance may have an increased odds of exhibiting histopathologic features associated with a greater risk of retinoblastoma progression, compared with private insurance (OR = 1.45 [0.73, 2.93]) [22].

### 3.5 Education Access and Quality

Four studies examined 16 associations within the domain of education access, attainment, and quality. Thirteen of the 16 associations (81.3%) were unfavorable, none were favorable, and 3 (18.7%) did not have directionality (‘NA’). Truong 2015 (moderate risk of bias) and Cheung 2013 (high risk of bias) found no meaningful difference in 5‐year survival (96.8% vs. 98.5%) [37] or retinoblastoma‐specific mortality (*p* = .41) [19] between low‐ and high‐educational attainment counties. Children living in counties with lower levels of education had higher odds of enucleation than children living in counties with higher levels of education (OR estimate range, stratified by race: 1.06 to 2.44) [37]. Truong 2015 also found that living in a county with lower educational attainment conferred higher odds of advanced disease at diagnosis both overall (aOR = 1.44 [1.01, 2.05]) [37]. Green 2016 (high risk of bias) also examined the association between higher levels of education in parental census tract zip‐codes and having high‐risk pathologic markers for retinoblastoma progression but found no evidence of difference across quartiles (effect size not reported, *p* = 0.08) [22]. Bunin 2011 (high risk of bias) studied the incidence of retinoblastoma and found no evidence of a reduction in the odds of developing retinoblastoma for higher versus lower educational attainment (aOR = 0.80 [0.40, 1.30]) [17].

### 3.6 Economic Stability

Eight studies examined a total of 63 associations within the domain of economic stability, including income, employment status, and occupational exposures in the years before conception. Thirty‐nine of the 63 associations (61.9%) were unfavorable, and 19 (30.2%) were favorable, 1 (1.6%) was null, and 4 (6.3%) were NA. We included parental occupational exposures under the economic stability domain, as parental work is tied to a family's economic stability. For mortality and survival, income levels did not appear to have an impact. Three studies found higher incomes may have decreased mortality risk compared with lower incomes [19, 30, 31]. Similarly, Truong 2015 (moderate risk of bias) found that poverty and unemployment showed a comparable relationship, with higher levels of both increasing the odds of enucleation overall (OR = 1.34 [0.98, 1.83] and OR = 1.21 [0.88, 1.66], respectively) and across all ethnic categories, except among Black individuals where greater poverty and unemployment measures resulted in a decreased odds of enucleation (OR = 0.68 [0.28, 1.60] and OR = 0.78 [0.34, 1.79], respectively). Truong 2015 and Green 2016 (high risk of bias) found poverty, unemployment, and income may have some relation with the extent of disease. Whereas Truong 2015 found higher poverty levels (aOR = 1.34 [0.94, 1.91]) and higher percentages of unemployment (aOR = 1.46 [1.02, 2.10]) overall were associated with greater odds of more advanced disease at diagnosis [37], Green 2016 found no clear relationship between decreasing income and high‐risk pathologic features of retinoblastoma (OR = 0.94 [0.51, 1.73]) [22].

Within this domain, incidence was primarily assessed in relation to occupational exposures, including welding fumes, sulfur dioxide, polycyclic aromatic hydrocarbons (as a group), ionizing radiation, paint, and chlorinated as well as non‐chlorinated volatile organic compounds [15, 33]. Of 29 occupational exposure associations assessed by Abdolahi 2013 (high risk of bias) and Omidakhsh 2018 (moderate risk of bias), there was no evidence of an association with retinoblastoma incidence in 28 [15, 33]. Bunin 2011 (high risk of bias) also assessed retinoblastoma incidence and found higher income may be associated with greater incidence of retinoblastoma (OR = 1.50 [0.70, 3.30]) [17].

### 3.7 Neighborhood and Built Environment

Overall, eight studies examined 292 associations within the built environment domain, all related to the incidence of retinoblastoma. Of the 292 associations, 177 (60.6%) were unfavorable, 102 (34.9%) were favorable, and 13 (4.5%) were null. Common exposures included tobacco as well as environmental exposures such as pesticides and industrial chemicals, rather than occupational as seen in the economic domain. Two studies found a mix of associations between retinoblastoma incidence and parental drug use (e.g., drinking and smoking) in the years preceding and during pregnancy [16, 23]. Whereas Azary 2016 (high risk of bias) did not find evidence of associations for maternal smoking before and during pregnancy [16], Heck 2016 (high risk of bias) estimated 9.4 and 3.0 times the odds of developing retinoblastoma bilaterally or unilaterally for mothers who smoked during pregnancy compared to mothers who did not [23]. Exposure to airborne chemicals and pesticides was assessed in six studies and the evidence was inconsistently associated with higher incidence of retinoblastoma [18, 34, 36, 38, 39, 40]. Heck 2015 [39] (low risk of bias) and Ghosh 2013 [38] (low risk of bias) found multiple unfavorable associations between prenatal exposure to pollutants such as ethyl benzene, chloroform, and nitric oxide, and the development of both unilateral and bilateral retinoblastoma, and other studies [18, 34, 36, 40] observed almost universally unfavorable associations.

### 3.8 Social and Community Context

Eleven studies examined 132 associations with retinoblastoma within the domain of social and community context. Of the 132 associations, 82 (62.1%) were unfavorable, 23 (17.4%) were favorable, 3 (2.3%) were null, and 24 (18.2%) were NA. Race or ethnicity was the most common exposure, assessed in 10 studies; other exposures included minority and language status, language isolation, immigration, crowding (i.e., people in the household), and gender. Three out of four studies found evidence that Black and ethnic minority participants had lower 5‐year survival rates than their White counterparts [19, 25, 37], although all four showed effect estimates that suggested an overall unfavorable association between minority status and higher mortality [19, 25, 31, 37]. Truong 2015 (moderate risk of bias) also found a worse 5‐year survival for immigrant children compared with non‐immigrant children (95.6% vs. 97.9%, *p* < 0.01) [37]. Holmes 2023 (moderate risk of bias) suggested that males may have a higher risk of mortality than females (aHR = 3.40 [0.30, 31.60]) [31]. Both Truong 2015 and Rajeshuni 2019 (moderate risk of bias) found identification with minority race or ethnicity was associated with increased odds of enucleation compared with White or non‐Hispanic populations [35, 37]. Truong 2015 also identified greater levels of household crowding as a factor that affects the likelihood of enucleation [37]. Similarly, Truong 2015 found that minority status, higher crowding, and higher levels of language isolation all carried higher odds of having more advanced disease at diagnosis [37]. Also, Green 2016 (high risk of bias) and Fei‐Zhang 2023 (moderate risk of bias) respectively found evidence that minority status and language isolation increased high‐risk pathologic findings and having advanced staging at presentation [22, 29]. Several studies found some evidence that minority groups had higher incidence of retinoblastoma compared with White non‐Hispanic populations [21, 27, 32], but this finding was not supported by all studies [17, 24].

### 3.9 Multiple Domains and Multiple Cancers

In addition to the above exposures that were related to single SDOH domains, 9 studies presented 27 associations for more complicated measures of exposure that span multiple domains, like socioeconomic deprivation, vulnerability, and index scores [29, 35, 37]. Twenty one of the 27 associations (77.8%) were unfavorable and 6 (22.2%) were favorable. As with the individual domains, multi‐domain factors that included components related to lower socioeconomic status (SES) generally had more unfavorable outcomes such as more advanced disease at diagnosis, greater likelihood of enucleation, and increased incidence of ocular neoplasm than components related to high SES [29, 35, 37].

Two studies examined multiple cancers, including uveal melanoma, conjunctival melanoma, and retinoblastoma. These studies did not separate results by cancer type and had a low prevalence of retinoblastoma; thus, their findings may not be comparable to the other studies included in this review [20, 28].

### 3.10 SDOH As the Outcome

Unlike most authors in our search, Reynolds 2021 (high risk of bias) evaluated the relationship between retinoblastoma treatment (receiving chemotherapy vs no chemotherapy and enucleation vs no enucleation) and education access and quality outcomes in 13 associations [26]. They found that children who received chemotherapy instead of enucleation were at greater odds of self‐reporting school difficulty (aOR = 5.40 [1.40, 21.70]) and needing an individualized education plan (OR = 11.50 [1.30, 98.10]) compared with those who were treated only with enucleation [26]. Similarly, among patients with unilateral retinoblastoma, a history of chemotherapy was associated with greater odds of self‐reported school difficulty (OR = 12.80 [1.50, 113.00]) compared with patients who did not undergo chemotherapy. They did not find any evidence of associations between receiving enucleation to treat retinoblastoma and the education‐related outcomes they investigated.

## 4 Discussion

We found 26 studies that had reported 552 associations between SDOH and retinoblastoma outcomes. Although the evidence for most associations was not sufficient to find an effect, the majority of estimates suggested that exposures related to lower SES such as lower income, lack of insurance, and rural residence tended to produce more unfavorable outcomes. This pattern was also observed for minority racial and ethnic groups compared to White non‐Hispanic groups. From a clinical and policy standpoint, the most actionable patterns were concentrated in social and community context, economic stability, and healthcare access and quality. Across these domains, minority race and ethnicity, language isolation, crowding, poverty, unemployment, and non‐private insurance were repeatedly associated with more advanced disease presentation, greater likelihood of enucleation, or worse survival. By contrast, the large body of studies on environmental and occupational exposures was most informative for disease cause and prevention, primarily through incidence outcomes, but less explicitly informative for immediate treatment delivery. Our displays of the associations (Figure 3) highlight the multi‐faceted nature of the SDOH‐retinoblastoma relationship and indicate that for most outcomes, no single social factor acts alone. Thus, addressing only one domain (income, e.g.,) is unlikely to be enough; instead, multi‐domain interventions are likely needed to successfully address variation in retinoblastoma health outcomes.

Existing evidence indicates that cancer outcomes are influenced by a multitude of factors, including genetic predisposition and environmental exposures. In the US, other recent reviews have noted gaps in ocular primary cancer outcomes, including inequities in hospital readmissions, advanced staging at diagnosis, likelihood of enucleation therapy, and mortality [28, 41]. Our review adds value beyond those summaries and beyond single‐database analyses because it synthesizes US evidence across all five Healthy People 2030 SDOH domains, integrates incidence, staging, treatment, and survival outcomes in one framework, and identifies which associations appear most consistently unfavorable across studies. It also brings together evidence from large registry studies and smaller causal studies of parental occupational and environmental exposures, which are less visible in routine surveillance datasets such as SEER. This synthesis helps distinguish potentially modifiable care‐related factors (e.g., insurance status, language isolation, access to specialized care) from exposures that are more relevant to long‐term prevention.

These findings reinforce the importance of considering SDOH in strategies aimed at reducing gaps in ocular cancer‐related health outcomes. Given these demonstrated associations, healthcare providers and policymakers should integrate SDOH considerations into their practices and policies. Specifically, socioeconomic status, access to optometric and to general and specialized ophthalmologic care, geographic location, and health literacy play critical roles in determining the timeliness of diagnosis and the effectiveness of treatment outcomes in retinoblastoma. Accordingly, targeted public health and health‐system responses should be designed not only to reduce broad barriers to care, but also to improve early detection and follow‐through among underserved groups. Such efforts could include improving communication with non‐English speakers and readers to encourage regular eye examinations, developing culturally relevant educational materials for parents, using telemedicine and other remote monitoring by specialized ophthalmologists in underserved areas, and implementing policies to reduce financial barriers to care. Further, incorporation of routine red‐reflex screening into access points outside of the pediatrician office, such as public health spaces, school‐based clinics, and school nursing services, may help reduce delays in presentation. Standardized referral pathways that minimize diagnostic delays, along with more direct supports such as patient navigation or transportation services, may also improve access to timely care and subsequent outcomes. Although retinoblastoma‐specific implementation evidence remains limited, these strategies have shown value in other pediatric cancer settings and may represent useful approaches for adaptation.

We also noted a handful of associations in which exposures typically viewed as harmful, like welding fumes, appeared to be associated with more favorable outcomes, such as reduced retinoblastoma incidence. Potential explanations for these paradoxical findings include residual confounding, exposure misclassification, the concept of hormesis, whereby low‐level exposure to an ordinarily harmful agent may trigger adaptive responses that improve certain aspects of health [42], and the “healthy worker effect,” in which individuals who remain employed may be inherently healthier than the general population due to factors such as baseline health or access to preventive health services [43]. Future studies of retinoblastoma should consider these phenomena when identifying controls for the groups evaluated and interpreting data, especially in analyses involving occupational or environmental exposures that appear protective.

Our review benefited from the quality and consistency of outcome measurement. Since retinoblastoma is a cancer diagnosis, many of the included studies utilized data from national databases (e.g., SEER, National Cancer Database) which employed standardized outcome definitions. This likely improved comparability for outcomes such as survival, mortality, and stage. However, that advantage did not extend to SDOH measurement or analytic strategy. Exposures were operationalized inconsistently across studies, and adjustment models varied even when investigators used the same underlying databases. As a result, standardized outcome definitions did not eliminate heterogeneity in the evidence base.

While conducting our review, we faced several challenges. One major challenge that precluded synthesis was the heterogeneity in study designs and the adjustment models used to control confounding. Studies that utilized the same datasets, such as SEER or the National Cancer Database, often used different methods to adjust for potential confounders and other variables, including race, age, and socioeconomic status. This inconsistency affects both the comparability of studies and the external validity of the evidence, as the differing adjustment models may have obscured true associations between SDOH and retinoblastoma. Another limitation is the lack of exploration of associations by laterality in the populations. Most studies reported the distributions of unilateral and bilateral retinoblastoma but did not stratify results accordingly. This approach may increase generalizability to retinoblastoma overall but limits inferences about how associations may differ between these disease states. For example, given the “save the life, globe, sight” treatment paradigm, enucleation decisions may differ between unilateral and bilateral cases, biasing observed associations.

## 5 Conclusion

Our systematic review highlights the association of different aspects of SDOH with retinoblastoma outcomes. In general, the directionality of associations was unfavorable for those with “worse” exposures compared with “better” exposures. Rather than pointing to a single dominant factor, the evidence suggests that retinoblastoma‐related disparities may arise across multiple, intersecting social and structural conditions. These findings support the need for more standardized SDOH measurement in retinoblastoma research and for health‐system and public health strategies that reduce barriers to timely diagnosis, referral, and treatment for underserved children and families.

## Author Contributions

**Vijay Joshi:** investigation; writing – original draft; methodology; validation; visualization; writing – review and editing; formal analysis. **Daniel Shaughnessy:** investigation; writing – original draft; methodology; validation; visualization; writing – review and editing; formal analysis; data curation. **Natalia Dellavalle:** investigation; writing – original draft; writing – review and editing; validation. **Louis Leslie:** investigation; writing – original draft; visualization; validation; methodology; writing – review and editing. **Michael Edwards:** methodology; investigation; writing – original draft; writing – review and editing. **Sandra Luna‐Fineman:** investigation; methodology; writing – original draft; writing – review and editing. **Timothy Waxweiler:** methodology; investigation; writing – original draft; writing – review and editing. **Tianjing Li:** conceptualization; funding acquisition; investigation; writing – original draft; methodology; writing – review and editing; project administration. **Riaz Qureshi:** conceptualization; investigation; writing – original draft; methodology; validation; writing – review and editing; visualization; project administration.

## Conflicts of Interest

The authors declare no conflicts of interest.

## Supporting information

Appendix_A.

Retino_PRISMA_2020_checklist.

Table_S1.

Table_S2.

## Data Availability

The data that supports the findings of this study are available in the supporting material of this article.
