# Supplementary material for: Influence of Social and Demographic Factors on Retinoblastoma Outcomes in the United States: A Systematic Review
Source: Cochrane Evid Synth Methods. 2026 May 16;4(3):e70081. doi: 10.1002/cesm.70081 (PMC13182604; doi:10.1002/cesm.70081)
Supplement: Supplementary file 1 — Appendix_A. [file CESM-4-e70081-s003.docx]

**APPENDIX A – Detailed methods for systematic review of social determinants of health and retinoblastoma**

We performed this review within a suite of systematic reviews being undertaken by Cochrane Eyes and Vision US Project (CEV@US) examining various aspects of eye health and their relation to the SDOH.^1^ In this review, we focused on retinoblastoma and followed a protocol that is published in full on Open Science Framework.^2^ No ammendments were made to the protocol. There are separate reviews that examine the relationship between SDOH and other relevant eye and vision topics and conditions such as dry eye, pediatric vision screening, telehealth, etc.^1-5^ A review by Korn et al. looked at the relationships between breast, cervical, colorectal, and lung cancer screening and SDOH, which provided a helpful model to set up our own literature search and overall approach in addition to the standard protocol established for the suite of reviews.^6^

**2.1 Eligibility criteria**

Study Design: Eligible studies were primary studies that examined the relationship between SDOH and retinoblastoma, utilized an observational or interventional design, were reported in English, and had findings published after 2000. Both quantitative and qualitative studies were eligible. We excluded studies revealing findings of biological and mechanistic causes of diseases. As an example, we would have excluded a study looking at genetic profiling of ocular neoplasms. We also excluded studies that were conducted exclusively in one subpopulation (restriction) without a comparison group. For example, we would have excluded a study that described retinoblastoma diagnosed in a children's hospital but did not compare this group with another group. Finally, we excluded studies that assessed the cost-effectiveness of different interventions.

Population: We included studies in populations with retinoblastoma. We excluded studies in populations of other ocular neoplasias (e.g., ocular melanoma), non-ocular neoplasias (e.g., cutaneous melanoma) that do not involve the eye or surrounding structures, as well as central nervous system tumors (e.g., pituitary adenomas, craniopharyngiomas, optic nerve sheath meningiomas) and skull base tumors (e.g., meningiomas, chordomas, chondrosarcomas, nasopharynx cancers) that can affect vision by compressing optic nerves, chiasm, or tracts. For studies that include both retinoblastoma and non-ocular neoplasms, we only included the study if data from the retinoblastoma subgroup was reported separately. We included participants from any study setting; for example, any relevant population-based, hospital/clinic-based, community-based, and school-based study was eligible. As our focus is on the US context because social determinants are highly culturally dependent, we only included studies conducted on (or including) US populations.

Exposure/Intervention(s): We included studies that focused on at least one SDOH within the five domains defined by the Healthy People 2030 framework^7^, as well as studies that investigated various indices as composite measures of SDOH (e.g., Yost index^8^). Associations may be multi-directional – thus we included studies when SDOH is used either as exposure or outcome. We included intervention studies that attempt to ameliorate SDOH. For example, we would have included a study that examined whether implementing community-based care in underserved areas influenced the stage at diagnosis of ocular neoplasms. We also included studies that examined whether SDOHs are barriers, facilitators, and/or moderators of retinoblastoma diagnosis and treatment effectiveness. For example, we would have included a study that looked at the relationship between food or housing insecurity and retinoblastoma treatment adherence. We excluded studies that included SDOH only as demographic or control variables. For example, if a study included race and ethnicity in a regression model, but the primary goal was not to examine the relationship between SDOH variables and retinoblastoma, this study would have been excluded.

Outcome: We did not exclude studies based on specific outcomes reported. As mentioned previously, the association could be bi-directional: we included studies relating SDOH to retinoblastoma (e.g., the effect of socioeconomic disparities on retinoblastoma survival) as well as studies relating retinoblastoma to SDOH (e.g., whether retinoblastoma diagnosis affects an individual's sense of social isolation or cohesion).

**2.2 Selection of studies**

For this review, we identified eligible studies from a master database on social determinants of ocular health that was developed and maintained by CEV@US. For the master database, a comprehensive literature search of Ovid MEDLINE, Embase, and Web of Science was conducted on November 23, 2024. Researchers from CEV@US pilot tested title and abstract screening and the full-text screening and tagging procedure before the formal review process began in Covidence. Two individuals worked individually in pairs to screen against the eligibility criteria for each title and abstract and full-text reports when deemed relevant. At the full text reviewing stage, the pair of researchers tagged each eligible record by eye condition and topic, SDOH domain, report type, study design, and age group. At both stages, we resolved discrepancies regarding eligibility for the master database through discussion and/or consultation with a third individual.

We selected all full-text reports tagged as 'ocular cancer' during the primary full-text screening stage for the master database. We included all types of ocular neoplasms (e.g., retinoblastoma, uveal melanoma, etc.) and neoplasms affecting the area around the eye or orbit (e.g., periocular cutaneous basal and squamous cell carcinoma). We also included the studies that grouped ocular and non-ocular neoplasms for further evaluation. Given the richness of associations we found, we focused this review solely on retinoblastoma and noted reports related to other ocular cancers for later use. For reviews and non-primary studies that satisfied the eligibility criteria for this review, we searched the reference lists for potentially eligible primary studies.

Two people worked independently to screen the studies selected addressing retinoblastoma for inclusion for this review. We documented reasons for exclusion. We generated a study flow diagram that describes the identification of studies. We resolved disagreements through discussion.

**2.3 Data extraction and risk of bias assessment**

We piloted a data extraction form using Systematic Review Data Repository (SRDR+) and Qualtrics. For each included study, one author extracted data and assessed the potential risk of bias, which a second author verified. We resolved discrepancies through discussion or adjudication by a third reviewer.

We extracted the following items related to the: (a) study (title, journal, author(s), year of publication, study objectives, design, sampling method, sample size participants in analysis, dates of follow-up, follow-up period as reported, and other information regarding where and how the participants were recruited), (b) population (eye condition and severity, age, sex, gender, race, ethnicity, and other demographic characteristics), (c) exposure (SDOH mapped to five Healthy People 2030 domains [economic stability, neighborhood and built environment, healthcare access and quality, education access and quality, and social and community context]^7^, constructs^1^, measures, and level of measurement, details of the interventions and factors relevant to implementation), (d) outcome (incidence, progression, and severity of retinoblastoma; visual impairment definition and frequency; treatment adherence; generic and vision-related quality of life; SDOH construct or variable (when used as outcomes)), (e) association (effect estimates (e.g., odds ratio) and associated measures of precision, and key findings and conclusions), and (f) risk of bias (see below).

Given the heterogenous set of study designs expected, we assessed the risk of bias using domains adapted from the Newcastle-Ottawa Scale.^9^ The Cochrane Eyes and Vision group has used this assessment for other systematic reviews in the broader social determinant of health project as it allows for a general measure of the potential for bias that is applicable to multiple different study designs and settings. The assessment includes five domain judgments and justifications, including: (1) Representativeness of the sample (in cross-sectional studies) or whether the study sample was free of selection bias (in other study designs), (2) Risk of information bias in the measurement of exposures, (3) Risk of information bias in the measurement of outcomes, (4) Risk of information bias due to missing data, and (5) Risk of bias due to confounding.

**2.4 Data synthesis**

We synthesized results using narratives, tables, and figures. We grouped the results by SDOH domain, population (e.g., ages, setting), and study design (e.g., observational, interventional). We anticipated a heterogenous set of studies that would not be amenable to meta-analysis. We used alternative methods such as Harvest plots and Sankey figures to guide a qualitative synthesis following the guidance described in Chapter 12 of the Cochrane Handbook.^10^

To help our qualitative synthesis, we classified each extracted association based on its statistical significance and direction of effect. Association directionality was classified as follows: favorable if "worse" exposure, compared with "better" exposure, was associated with improved outcomes (e.g., reduced incidence); unfavorable if "worse" exposure was linked to worse outcomes (e.g., increased mortality or extreme treatments like enucleation); and null if no clear relationship was observed. For instance, if a study found that lower income levels were associated with increased mortality compared with higher income levels, we classified this as unfavorable. This classification logic was applied consistently across all exposures, including distance to agriculture (closer vs. further away), insurance type (public or none vs. private), chemical exposure (any vs. none), and race/ethnicity (any minority group vs. majority group). In some cases, the directionality of the exposure-outcome relationship could not be determined (e.g., lack of a clearly defined reference group or multi-group tests for homogeneity that did not yield a single comparison group), and these associations were labeled "NA" to avoid erroneous assignment of favorable or unfavorable status. For example, an analysis that evaluated poverty level by race and ethnicity in four groups (White non-Hispanic, White Hispanic, Black, and Others) via a Mantel-Haenszel test for homogeneity that used no definitive reference category was classified as "NA." For the studies that only reported P-values and did not report effect estimates for their associations, we classified the direction as "Not applicable".

We identified a variety of outcome types, including retinoblastoma incidence, effects of treatment and care that patients received, and mortality/survival. We classified the outcomes into one of the following general categories to organize our synthesis: adherence to cancer surveillance, cancer-specific or all-cause mortality, cancer survival, developmental delay/school difficulties, diagnosis and staging of cancer, incidence of childhood retinoblastoma, radiation treatment side effects, and type of ocular cancer treatment. We organized our results to present associations for the outcomes in the following order, where reported: mortality or survival, type of treatment, having advanced disease at diagnosis, and incidence of retinoblastoma. Because no meta‑analysis was undertaken, sensitivity analyses of pooled effect estimates were not applicable. Additionally, per the protocol, we did not conduct assesments for reporting bias or certainty of evidence.^2^

**References**

1. Li T, Abraham, A., Collins, M., Ehrlich, J.R., Elam, A., Lamoureux E., Piper C., Summers A. Social determinants of eye health in the United States: a systematic review protocol. 2023;doi:10.17605/OSF.IO/M8B4Q

2. Joshi V* SD, Dellavalle N, Leslie L, Edwards M, Luna-Fineman S, Waxweiler T, Hawkins B, Li T, Qureshi R. . Social determinants of ocular neoplasia in the United States: a systematic review protocol. *Open Science Framework*. February 6, 2024 2024;doi:<https://doi.org/10.17605/OSF.IO/6JSPZ>

3. Liu SH, Shaughnessy D, Leslie L, et al. Social Determinants of Dry Eye in the United States: A Systematic Review. *Am J Ophthalmol*. May 2024;261:36-53. doi:10.1016/j.ajo.2024.01.015

4. John Gorham AdAC, Angell Shi, Louis Leslie, Anne Lynch, Nicholas Quan, Tianjing Li. Social determinants of retinopathy of prematurity in the United States: a systematic review protocol. Protocol. *Open Science Framework*. January 22, 2024 2024;doi:<https://doi.org/10.17605/OSF.IO/GEJT7>

5. Choo A. LL, Tzang C., Liu S., Li T., Kuo I. Social determinants of cataract surgery in the United States: a systematic review protocol. *Open Science Framework*. 2024;doi:10.17605/OSF.IO/2X3WK

6. Korn AR, Walsh-Bailey C, Correa-Mendez M, et al. Social determinants of health and US cancer screening interventions: A systematic review. *CA Cancer J Clin*. Sep-Oct 2023;73(5):461-479. doi:10.3322/caac.21801

7. Social determinants of health. U.S. Department of Health and Human Services. Accessed September 22, 2023. <https://health.gov/healthypeople/priority-areas/social-determinants-health>

8. Yost K, Perkins C, Cohen R, Morris C, Wright W. Socioeconomic status and breast cancer incidence in California for different race/ethnic groups. *Cancer Causes Control*. Oct 2001;12(8):703-11. doi:10.1023/a:1011240019516

9. GA Wells BS, D O'Connell, J Peterson, V Welch, M Losos, P Tugwell,. The Newcastle-Ottawa Scale (NOS) for assessing the quality of nonrandomised studies in meta-analyses. Accessed July 11, 2022. <https://www.ohri.ca/programs/clinical_epidemiology/oxford.asp>

10. McKenzie JE BS. Chapter 12: Synthesizing and presenting findings using other methods [last updated October 2019]. In: Higgins JPT TJ, Chandler J, Cumpston M, Li T, Page MJ, Welch VA, ed. *Cochrane Handbook for Systematic Reviews of Interventions version 65*. Cochrane; 2024:chap 12.
